# From recanalization to recovery: inflammation and futile reperfusion after mechanical thrombectomy

**DOI:** 10.3389/fneur.2026.1950009

**Published:** 2026-10-13

**Authors:** Furong Zhang, Kai Wang, Xinfang Zhang, Qing Zhao

**Affiliations:** Department of Cerebrovascular Surgery, Heze Hospital Affiliated to Shandong First Medical University, Heze, China

**Keywords:** acute ischemic stroke, blood–brain barrier, futile reperfusion, inflammation, mechanical thrombectomy, neurovascular unit, neutrophil-to-lymphocyte ratio

## Abstract

Mechanical thrombectomy (MT) has markedly improved reperfusion outcomes in patients with acute ischemic stroke caused by large-vessel occlusion. However, successful angiographic reperfusion does not invariably translate into functional independence. Poor functional outcome despite successful angiographic reperfusion is commonly described as futile reperfusion. This clinical-angiographic mismatch may reflect persistent tissue injury, including inflammatory injury, alongside pretreatment damage and other determinants of outcome. Experimental findings and clinical associations implicate persistent inflammatory activation, thrombo-inflammation, endothelial dysfunction, microvascular no-reflow, and blood–brain barrier disruption as contributors to secondary tissue injury after MT. Here, we examine potential inflammatory contributions to futile reperfusion and consider how inflammatory biomarkers might inform risk stratification after MT. Ischemia activates microglia, endothelial cells, platelets, complement pathways, and circulating immune cells. Subsequent reperfusion may amplify oxidative stress, cytokine signaling, neutrophil-endothelial interactions, neutrophil extracellular trap formation, matrix metalloproteinase activity, and barrier failure. Together, these processes can promote microvascular obstruction, cerebral edema, hemorrhagic transformation, infarct growth, and impaired neurological recovery despite successful angiographic reperfusion. Among circulating biomarkers, the neutrophil-to-lymphocyte ratio has been studied repeatedly, alongside platelet-to-lymphocyte ratio, systemic immune-inflammation index, systemic inflammation response index, interleukin-6, matrix metalloproteinase-9, and other molecular markers. Their specificity, incremental predictive value, and clinical utility require further validation in standardized thrombectomy cohorts. We therefore view inflammation as a plausible biological link between macrovascular reperfusion and persistent tissue-level injury after MT. From a translational perspective, the immediate priorities are to standardize sampling protocols, characterize dynamic inflammatory trajectories, develop multimodal prediction models, and test biomarker-guided therapeutic strategies.

## 1 Introduction

Evidence for mechanical thrombectomy (MT) in acute ischemic stroke (AIS) due to large-vessel occlusion (LVO) includes the initial randomized trials (1–7), later-window trials (8, 9), and trials in patients with large infarct cores (10, 11). Meta-analytic evidence and guidelines support MT in selected patients (12, 13), but angiographic success does not guarantee functional recovery.

Recanalization means reopening an occluded artery; angiographic reperfusion describes restored downstream flow (14). Here, “futile reperfusion” denotes poor functional outcome despite successful angiographic reperfusion, principally 90-day modified Rankin Scale (mRS) scores of 3–6 after modified Thrombolysis in Cerebral Infarction (mTICI) grades 2b-3. This operational definition is not universal, and mTICI 2b-3 does not establish complete or adequate tissue reperfusion. “Futile recanalization” describes overlapping clinical endpoints in the literature (15–18), although arterial reopening and downstream reperfusion differ biologically. We retain each study’s angiographic scale, including expanded Thrombolysis in Cerebral Infarction (eTICI), threshold, functional-outcome definition, and follow-up time.

Age, baseline National Institutes of Health Stroke Scale (NIHSS) score, prestroke disability, and procedural factors explain part of outcome heterogeneity (15, 16). Structural injury, microvascular failure, and collateral supply also influence recovery (17–20), leaving a potential role for peri-reperfusion biology.

Ischemia and reperfusion activate immune responses alongside oxidative and excitotoxic pathways (21–23), while neuronal and neurovascular injury amplifies inflammatory signaling (24–26). Microglia and infiltrating immune cells contribute to inflammation (27–30), with lymphocyte and microglial effects depending on context (31, 32). Systemic inflammatory signals and neutrophil recruitment can worsen injury (33–35), and platelet-immune interactions contribute to thrombo-inflammation (36–39). These processes may impair microvascular perfusion and disrupt the blood–brain barrier (BBB).

This review examines inflammatory mechanisms and circulating biomarkers in relation to futile reperfusion after MT, distinguishing experimental findings from clinical associations and considering implications for future prediction and treatment studies.

Futile reperfusion does not establish that MT provided no benefit. Microvascular no-reflow is impaired microvascular perfusion despite arterial reopening (40), not a synonym for poor outcome. Poor outcome alone establishes neither no-reflow nor inflammatory injury. Recanalization, angiographic and tissue reperfusion, infarct evolution, and functional outcome remain distinct endpoints.

### 1.1 Literature search and scope

This narrative review was informed by searches of PubMed/MEDLINE, Embase, and Web of Science from database inception through July 2026. Search terms combined controlled vocabulary, where available, and free-text variants for acute ischemic stroke, large-vessel occlusion, mechanical thrombectomy, successful reperfusion or recanalization, futile reperfusion or futile recanalization, inflammation, inflammatory biomarkers, the blood–brain barrier, and microvascular no-reflow. Reference lists of relevant reviews and included reports were also screened, and the searches were updated through July 2026. We prioritized English-language studies involving adults with AIS due to LVO who were treated with MT, including randomized trials, prospective and retrospective clinical cohorts, systematic reviews, meta-analyses, and guideline statements that reported angiographic, tissue-level, or functional outcomes after reperfusion. Duplicate or overlapping cohorts were retained only when they provided distinct sampling times, biomarkers, outcomes, or adjusted analyses; otherwise, the most complete or most recent report was used. The synthesis distinguishes clinical MT cohorts and trials, ex vivo studies of MT-retrieved material, broader AIS or thrombolysis cohorts, and animal or cellular models. These settings differ in applicability to patients after MT. Observational associations, experimental interventions, and randomized clinical findings also support different levels of causal inference. This article was designed as a narrative review and did not follow a prespecified systematic-review protocol.

Table 1 is a non-exhaustive selection of original clinical studies, prioritizing poor functional outcome despite successful angiographic reperfusion. Selected broader MT cohorts illustrate other biomarkers, serial measurements, or local vascular sampling. Studies were chosen for clinical relevance and sufficiently detailed sampling, endpoint, and adjusted-estimate reporting to permit interpretation. Both significant and nonsignificant findings are included, and study-specific definitions and cohorts not restricted to successful reperfusion are identified.

**Table 1:** Representative clinical evidence for inflammatory biomarkers after mechanical thrombectomy.

Study, design and sample	Marker, blood source and timing	Success-only cohort and endpoint	Selected estimate (95% CI)	Covariates in the displayed model	Interpretation and confounding
Lattanzi et al., 2021 (82). Single-center retrospective cohort; *n* = 184.	SIRI; peripheral blood at admission, within 24 h of onset. Venous/arterial source not specified.	Yes: TICI 2b/3. Three-month mRS ≥ 3.	Per 1-unit SIRI increase: aOR 1.08 (1.01–1.17).	Age, sex, hypertension, baseline NIHSS and ASPECTS.	Modest discrimination; no external validation. Etiology and infection were absent from the displayed model.
Feng et al., 2022 (74). Single-center retrospective analysis of prospectively collected data; *n* = 170.	NLR at admission, within 24 h and 3–7 days after EVT; blood compartment unspecified.	Yes: mTICI 3 only. Ninety-day mRS 3–6.	Per 1-unit NLR increase: within 24 h, aOR 1.070 (1.012–1.131); at 3–7 days, 1.129 (1.032–1.236), in separate models.	Age, diabetes, CAD, baseline NIHSS, collateral status, glucose and general anesthesia.	Admission NLR was not significant after adjustment. Etiology and infection were absent from these models.
Guan et al., 2023 (86). Single-center retrospective cohort; *n* = 101.	Admission LMR; blood compartment and precise sampling interval unspecified.	Yes: mTICI ≥2b. Ninety-day mRS 3–6.	Per 1-unit LMR increase: aOR 0.64 (0.412–0.984).	Age, ASPECTS, baseline NIHSS, baseline systolic pressure, hypertension, NLR, PLR and sICH.	Model includes a post-treatment complication; no external validation. NLR/PLR were not significant in this model.
Cao et al., 2024 (76) Single-center retrospective cohort; *n* = 482.	NLR, PLR, SII, SIRI; peripheral venous blood at admission and ≤24 h after recanalization.	Yes: mTICI 2b–3. 90-day mRS 3–6.	Day 1, per raw unit, aOR: NLR 1.061 (1.022–1.102), Pc = 0.024; PLR 1.002 (1.001–1.004), Pc = 0.058; SII 1.000 (1.000–1.000), Pc = 0.009; SIRI 1.092 (1.018–1.172), Pc = 0.157.	Age, sex, diabetes, CAD, drinking, prior stroke, antiplatelet use, admission NIHSS/ASPECTS, puncture-to-recanalization time, anesthesia, passes, mTICI and sICH.	PLR/SIRI associations did not survive Bonferroni correction. Recent/concurrent infection excluded; TOAST not adjusted. SII rounded in source.
Chen et al., 2024 (80) Prospective nationwide EVT registry analysis; *n* = 1,002.	SII (P × N/L/100); first blood test at admission, before EVT. Sampling site unspecified.	Yes: mTICI 2b–3 and arterial patency at 24 h. 90-day mRS 3–6.	Model 3, per 1-SD SII increase (SD = 12): aOR 1.223 (1.053–1.444).	Age, sex, CAD, AF, current smoking, baseline NIHSS/ASPECTS, serum glucose, onset-to-recanalization time, thrombectomy passes, occlusion location and TOAST subtype.	TOAST adjusted. Peristroke infection not collected; collaterals unadjusted. EVT cohort not restricted to MT.
Han et al., 2025 (81) Single-center retrospective cohort; *n* = 741.	SII ≤ 24 h after EVT (Methods). Blood compartment unspecified.	Yes: mTICI 2b/3. 3-month mRS 3–6.	Per 1-unit ln(SII): aOR 1.29 (1.04–1.60), from the sensitivity model adding baseline NIHSS.	Age, sex, smoking, hypertension, diabetes, AF, IVT, ASPECTS, puncture-to-reperfusion time and baseline NIHSS.	TOAST examined in subgroups, not adjusted. Pneumonia not systematically collected.
Mechtouff et al., 2021 (69) Single-center HIBISCUS-STROKE cohort; *n* = 164 overall, 133 in futility analysis.	Peripheral serum IL-6 at admission (before IVT), 6, 24 and 48 h, and 3 months from admission.	Yes, futility analysis: TICI 2b/3. 3-month mRS 3–6.	High vs. low 24-h IL-6 (median split): aOR 6.15 (1.71–22.10). Numeric cutoff not reported.	Table 2 footnote: age, baseline NIHSS, hypertension, IVT, intracranial ICA occlusion and infarct growth.	Etiology not adjusted. Admission antibiotic use/inflammatory disease excluded; no infection covariate. Model includes infarct growth.
Gao et al., 2024 (73) Single-center retrospective cohort; *n* = 423.	NLR; venous blood at admission, 24 h and 72 h after MT.	No; mTICI 2b–3 reported. 90-day mRS 3–6.	Per 1-unit NLR, aOR: admission 1.12 (1.06–1.19); 24 h 1.15 (1.10–1.20); 72 h 1.38 (1.28–1.49).	Age, hyperlipidemia, AF, baseline NIHSS/GCS and thrombectomy attempts.	Admission infection excluded; etiology not adjusted. Exclusion of early deaths may bias serial estimates.
Prandin et al., 2025 (71) Four-center retrospective cohort; *n* = 970.	NLR; venous blood at admission and 24 h. Exact relation to reperfusion is unclear.	No; success defined as mTICI 2b/2c/3. Favorable mRS 0–2 at 90 ± 14 days.	Per 1-unit 24-h NLR: aOR 0.909 (0.850–0.972) for favorable outcome.	Age, hypertension, hypercholesterolemia, AF, diabetes, prior stroke/TIA, smoking, ASPECTS, admission NIHSS, general anesthesia, glucose, hemoglobin, onset-to-needle time, sICH, 24-h CRP and angiographic success.	Infection at admission or within 48 h excluded. Etiology not adjusted. Model includes post-treatment variables.
Kollikowski et al., 2024 (68) Prospective single-center cross-sectional study; *n* = 132.	MMP-9; intracranial collateral blood before recanalization, paired with systemic arterial samples.	No; success defined as eTICI ≥2b50. Discharge mRS ≥ 5.	Local MMP-9 per 1,000 ng/mL: aOR 2.33 (1.068–5.1).	Baseline NIHSS/ASPECTS, admission systolic pressure and lack of reperfusion (retained model).	Etiology/infection absent from this model. Discharge disability is a different endpoint from 90-day futility.

Scope and selection. This non-exhaustive narrative selection prioritizes studies of poor outcome after successful reperfusion, with selected broader cohorts providing complementary biomarker and sampling evidence (Section 1.1). Original reperfusion definitions and endpoints are retained. Angiographic success does not establish adequate tissue perfusion. Estimates. Pc denotes the Bonferroni-corrected *p* value. Rounding prevents reliable rescaling of the SII estimate in Cao et al. (76); the reported value should not be interpreted as a null effect. ORs cannot be compared directly when their scales or outcome directions differ. SIRI in Lattanzi et al. is expressed in 10^9^/L; the reported unit increment uses this scale. Interpretation. Sampling windows vary by study. These observational associations neither establish causality nor define validated clinical phenotypes. Systemic biomarkers are nonspecific. Excluding infection at baseline does not eliminate confounding from subsequent pneumonia, and adjusting for AF does not replace adjustment for stroke etiology. AF, atrial fibrillation; aOR, adjusted odds ratio; ASPECTS, Alberta Stroke Program Early Computed Tomography Score; CAD, coronary artery disease; CI, confidence interval; CRP, C-reactive protein; TICI, Thrombolysis in Cerebral Infarction; mTICI, modified Thrombolysis in Cerebral Infarction; eTICI, expanded Thrombolysis in Cerebral Infarction; EVT, endovascular treatment; GCS, Glasgow Coma Scale; ICA, internal carotid artery; IL-6, interleukin-6; IVT, intravenous thrombolysis; ln, natural logarithm; LMR, lymphocyte-to-monocyte ratio; MMP-9, matrix metalloproteinase-9; mRS, modified Rankin Scale; MT, mechanical thrombectomy; NIHSS, National Institutes of Health Stroke Scale; NLR, neutrophil-to-lymphocyte ratio; OR, odds ratio; PLR, platelet-to-lymphocyte ratio; SD, standard deviation; sICH, symptomatic intracranial hemorrhage; SII, systemic immune-inflammation index; SIRI, systemic inflammation response index; TIA, transient ischemic attack; TOAST, Trial of Org 10,172 in Acute Stroke Treatment; P/N/L, platelet/neutrophil/lymphocyte counts.

## 2 Potential inflammatory mechanisms contributing to futile reperfusion after MT

### 2.1 Ischemic inflammatory priming: establishing susceptibility to futile reperfusion

Inflammatory activation begins after arterial occlusion and shapes the biological environment in which reperfusion occurs. Injured brain cells release damage-associated molecular patterns (DAMPs), including extracellular peroxiredoxins. In mice, these proteins activated Toll-like receptor 2 and 4 signaling in macrophages, and antibody neutralization reduced inflammatory responses and ischemic injury (41).

Microglia are among the earliest responders (28, 32). Their effects depend on context: selective microglial depletion increased ischemic injury in mice (42), while delayed interleukin-17-producing lymphocyte responses contributed to injury in another experimental study (31). At the vascular interface, imaging in rats showed leukocyte margination and fibrinogen deposition in cortical venules during arterial occlusion, with thrombi persisting after recanalization (43). These findings support early thrombo-inflammation, but also show that immune-cell responses are not uniformly injurious.

This early inflammatory priming may be clinically important because MT restores macrovascular flow into a tissue bed with pre-existing injury. Successful angiographic reperfusion may therefore coexist with impaired microvascular flow. The degree of inflammatory priming may help explain why patients with similar occlusion sites and angiographic results have different outcomes, although this remains a mechanistic hypothesis after human MT.

Collateral circulation provides additional context. Clinical angiographic evidence links pial arterial filling to outcome (20), but does not establish that collateral status determines endothelial activation or leukocyte recruitment. Whether collateral supply modifies inflammatory priming remains uncertain.

Systemic inflammation may further modify this primed state. In experimental stroke, systemic inflammatory stimulation worsened injury through interleukin-1- and neutrophil-dependent mechanisms (33) and impaired tissue reperfusion through endothelin-dependent mechanisms (44). The contribution of these mechanisms to outcomes after human MT remains to be established.

### 2.2 Reperfusion-triggered thrombo-inflammation and microvascular failure

Mechanical thrombectomy rapidly restores arterial patency, but reperfusion itself may intensify injury. Experimental ischemia–reperfusion studies linked succinate oxidation to mitochondrial reactive oxygen species (ROS) generation (45). In cultured brain endothelial cells and mice, inhibition of the NLR-family pyrin domain-containing protein 3 (NLRP3) inflammasome reduced endothelial death and preserved BBB integrity (46). In a mouse model with thrombolytic recanalization, neutrophil obstruction impaired capillary perfusion, and neutrophil depletion improved microvascular reperfusion (47). These findings require validation after human MT.

Neutrophil extracellular traps (NETs) provide a potential link between immune activation and thrombosis. NETs were identified in retrieved human thrombi, and deoxyribonuclease I (DNase I) enhanced tissue-type plasminogen activator (tPA)-mediated lysis ex vivo (48, 49). In mice, platelet-specific deletion of high mobility group box 1 reduced NET formation and brain injury (50). Human clot findings do not directly demonstrate downstream capillary obstruction, while the platelet–neutrophil mechanism remains experimental.

Thus, futile reperfusion may involve a mismatch between restored arterial flow and persistent tissue injury, alongside pretreatment damage and other determinants. Experimental no-reflow and clinical perfusion imaging distinguish arterial reopening from tissue reperfusion (40, 51). In mice, sustained pericyte contraction restricted capillary flow despite arterial reopening (52); its contribution to outcomes after human MT remains uncertain.

Thrombectomy may also introduce procedure-related inflammatory stress. NET content in retrieved thrombi was associated with longer procedures and more device passes (49), but these associations do not establish that procedural manipulation causes inflammation or futile reperfusion.

Complement activation provides another potential link between ischemia–reperfusion and injury. Targeting mannose-binding lectin (MBL) reduced injury in rodent cerebral ischemia models (53). Complement-targeted strategies remain experimental, with no established benefit after MT.

Stroke etiology may influence thrombo-inflammation through differences in thrombus formation, clot composition, immune activation, and vascular disease. Findings in large-artery atherosclerosis and cardioembolism are inconsistent: Laridan et al. found more citrullinated histone H3 staining in cardioembolic thrombi (48), whereas Ducroux et al. found no association between NET content and etiology (49). Cell abundance does not establish activation state or justify ranking etiologies by inflammatory activity. Whether these differences contribute to futile reperfusion requires direct links between clot findings, downstream perfusion, and functional outcome.

### 2.3 Microvascular no-reflow, endothelial dysfunction, and BBB disruption

Cerebral no-reflow was originally described as incomplete restoration of microvascular perfusion after transient ischemia (40). In contemporary stroke practice, postrecanalization perfusion deficit has re-emerged as a candidate explanation for incomplete recovery after successful angiographic reperfusion following MT (18, 54–56). Mechanisms proposed from experimental studies and broader stroke literature include stalled neutrophils, endothelial swelling, pericyte constriction, platelet-fibrin microthrombi, vasoconstrictive signaling, and extravascular compression by edema (44, 47, 56, 57).

Endothelial dysfunction is central to this process. Ischemia–reperfusion disrupts endothelial tight junctions and the extracellular matrix supporting the neurovascular unit (58–60). Experimental and clinical studies link matrix metalloproteinases to vascular injury and hemorrhagic complications (61–63). Tissue-type plasminogen activator-linked barrier opening and broader neuroinflammatory pathways reinforce this vulnerability (64, 65). BBB disruption therefore acts as both a consequence of reperfusion injury and a mediator of further tissue damage.

Matrix metalloproteinase-9 (MMP-9) is especially relevant because it links inflammation with BBB failure. Experimental work supports a contribution of MMP-9 to thrombolysis-associated hemorrhagic transformation in rats (63), while increased MMP-9 expression in human stroke tissue provides observational evidence (61). Reviews summarize its matrix- and barrier-related actions (59). Human thrombolysis studies report prognostic associations (62), and experimental work demonstrates tPA-linked barrier opening (64); these findings should not be treated as equivalent evidence of causality. In MT cohorts, circulating or locally sampled MMP-9 has been associated with infarct growth, hemorrhagic transformation, or poor outcome (66–68). Thus, the mechanistic evidence and the MT-population evidence support different levels of inference and should not be conflated.

The clinical challenge is that standard angiography is relatively insensitive to these tissue-level events. Even a high mTICI grade does not exclude patchy capillary obstruction, delayed hypoperfusion, impaired venous outflow, or BBB leakage. Perfusion imaging, permeability imaging, susceptibility imaging, and infarct-growth assessment may therefore be necessary to characterize tissue perfusion and injury more precisely.

This distinction also has implications for endpoint selection. Trials and observational studies that use only final mTICI grade may underestimate the contribution of microvascular pathology. Combining mTICI with early perfusion normalization, infarct expansion, edema formation, and BBB permeability could test whether inflammatory measurements are associated with impaired tissue reperfusion and subsequent injury (Figure 1).

**Figure 1:**
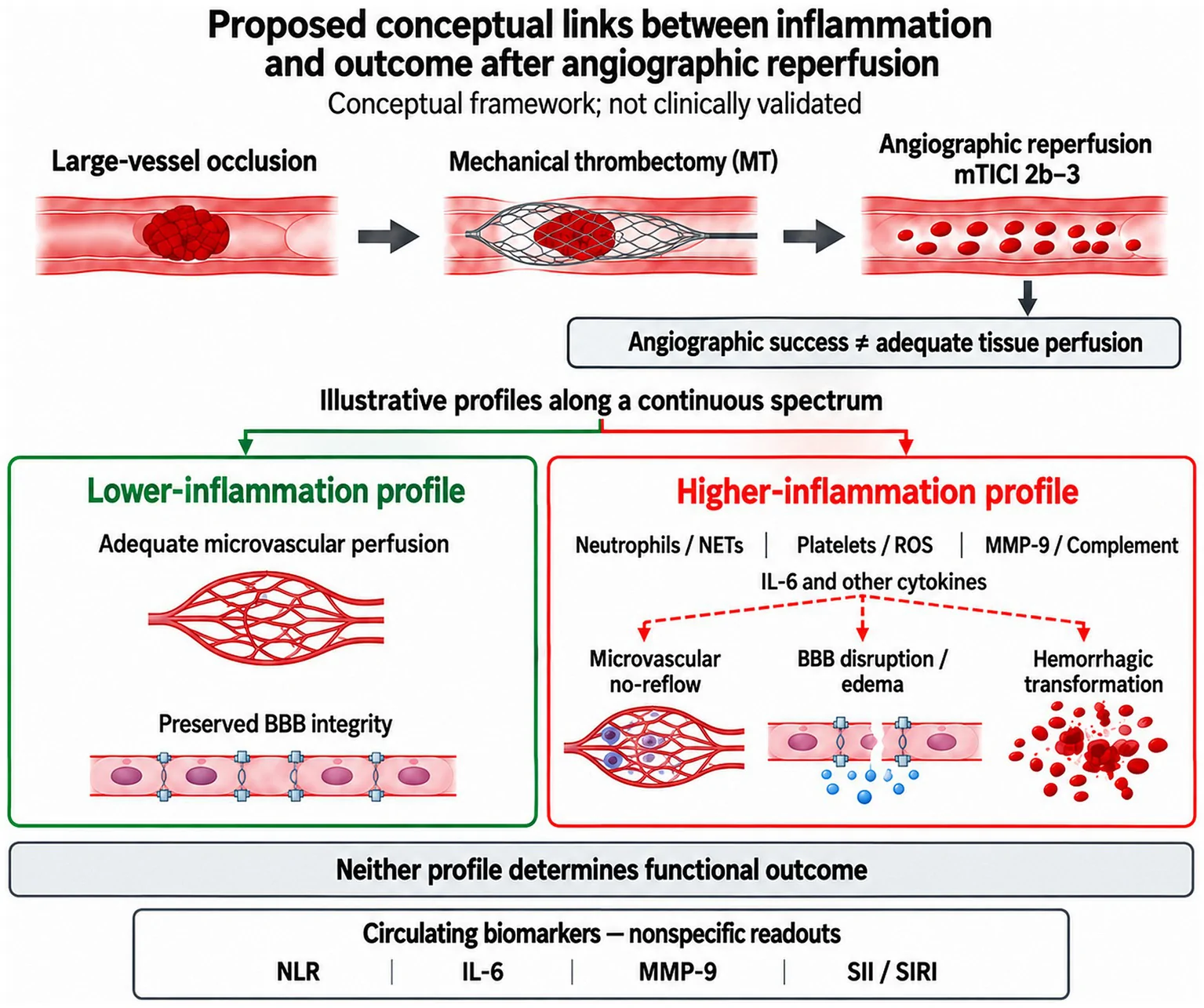
Proposed links between inflammation and outcome after angiographic reperfusion. Mechanical thrombectomy (MT) reopens the occluded artery, but angiographic reperfusion does not ensure adequate tissue perfusion. The lower- and higher-inflammation profiles illustrate possible patterns within a heterogeneous spectrum; they are not validated clinical categories. Dashed arrows mark proposed links drawn from experimental and clinical studies with differing strength of causal evidence. Neither profile determines functional outcome, which also depends on pretreatment tissue damage and other factors. Poor outcome alone cannot establish no-reflow or inflammatory injury. Circulating biomarkers are nonspecific and cannot independently establish cerebral inflammation, local NET formation or no-reflow. The framework offers no validated thresholds or basis for changing treatment or monitoring. BBB, blood–brain barrier; NETs, neutrophil extracellular traps; ROS, reactive oxygen species; MMP-9, matrix metalloproteinase-9; IL-6, interleukin-6; NLR, neutrophil-to-lymphocyte ratio; SII, systemic immune-inflammation index; SIRI, systemic inflammation response index; mTICI, modified Thrombolysis in Cerebral Infarction.

## 3 Inflammatory biomarkers and outcomes after MT

Routine angiography cannot directly measure tissue-level inflammatory injury, BBB integrity, or microvascular perfusion after MT. Circulating biomarkers may therefore provide complementary information by reflecting systemic responses and injury processes not captured by mTICI grading alone. Such measurements do not independently identify cerebral inflammation or its causal contribution. Their clinical value, however, depends on whether they improve risk stratification beyond established clinical, imaging, and procedural predictors.

A useful biomarker after MT should satisfy several criteria. It should be biologically plausible, measurable in a clinically relevant time window, reproducible across laboratories, independent of obvious confounders, and additive to existing clinical and imaging models. Most current inflammatory markers meet the first two criteria, but many still require stronger evidence for standardization, incremental predictive value, and clinical actionability.

Timing is central. Pre-reperfusion measurements may reflect pre-existing systemic inflammation as well as the response to ongoing ischemia; an admission sample is not necessarily a verified pre-reperfusion sample. Early post-MT changes may include responses to ischemia, reperfusion, and procedural stress, while persistent elevations over subsequent days may accompany infarct growth, microvascular dysfunction, infection, or systemic complications. These are overlapping processes, not fixed biological phases. Serial blood measurements should therefore be interpreted alongside the timing of perfusion abnormalities, BBB disruption, infarct evolution, and later functional outcome. A later association alone does not establish that inflammation caused injury or that a trajectory improves prediction beyond baseline information. Representative study-level evidence is summarized in Table 1.

Admission, immediate postprocedural, 6-h, 24-h, and day-3 sampling are suggested windows for longitudinal research; no clinical consensus supports this schedule. Each interval needs a defined starting point, such as the final angiographic run for postprocedural samples. Time from stroke onset and time from the procedure to sampling should be recorded separately. Published sampling schedules should retain their original starting points. HIBISCUS-STROKE, for example, measured interleukin-6 (IL-6) at admission and at 6, 24, and 48 h and 3 months from admission (69), rather than from reperfusion. Prospective studies are needed to assess the feasibility and added value of the proposed schedule.

A 2026 systematic review and meta-analysis included 37 studies and 8,780 patients treated with reperfusion therapy, but only 10 studies contributed to the quantitative synthesis, and all pooled analyses involved intravenous thrombolysis cohorts. Ten studies of MT were identified but were too heterogeneous in design, biomarker assessment, and outcome reporting for meta-analysis (70). Thus, this synthesis of prognostic associations warrants caution: MT-specific biomarker conclusions remain limited and heterogeneous.

### 3.1 Neutrophil-to-lymphocyte ratio

The neutrophil-to-lymphocyte ratio (NLR) is a frequently studied inflammatory biomarker in patients undergoing MT. Calculated as neutrophil count divided by lymphocyte count from the complete blood count (CBC), NLR may reflect systemic inflammatory and stress responses. A high NLR does not directly measure neutrophil activation, NET formation, or microvascular injury, and a low lymphocyte count does not independently establish adaptive immune dysfunction (71–73).

MT cohorts have associated higher NLR at admission or later sampling times with poor functional outcome and selected complications (71–73). In a retrospective multicenter cohort of 970 patients, Prandin et al. assessed admission and 24-h samples in relation to 90-day outcome (71). Gao et al. assessed admission, 24-h, and 72-h post-MT values in 423 patients and reported stronger associations for day-3 indices (73). These broader MT cohorts should not automatically be interpreted as cohorts restricted to successful reperfusion. Stronger performance at a later time point also does not establish the added predictive value of serial trajectories over a model using information already available at that time.

Evidence from cohorts restricted to angiographic success also depends on sampling time. Among 170 patients with complete reperfusion (mTICI 3), Feng et al. found that NLR within 24 h and at 3–7 days after endovascular treatment (EVT) was associated with 90-day mRS 3–6, whereas admission NLR was not significant after adjustment (74). In 456 successfully reperfused patients, Xu et al. likewise reported an adjusted association for postoperative NLR, but not admission NLR (75). These observational findings do not establish that serial measurements improve prediction beyond established models.

NLR should not be interpreted as a specific marker of cerebral inflammation. Infection, age, comorbid inflammatory disease, corticosteroids, stress physiology, and treatment-related factors can alter leukocyte profiles. Serial trajectories and integration with clinical and imaging models require prospective assessment rather than reliance on isolated cutoff values.

One practical advantage of NLR is its availability in virtually all stroke centers. This makes it attractive for large registries, quality-improvement datasets, and pragmatic prediction tools. However, accessibility should not be mistaken for specificity. NLR is best interpreted as a marker of systemic inflammatory burden rather than as direct proof of intracerebral inflammation.

### 3.2 Platelet-to-lymphocyte ratio

The platelet-to-lymphocyte ratio (PLR) provides complementary information by combining platelet and lymphocyte counts, but does not directly measure platelet activation. Experimental platelet-dependent NET formation in mice (50) and NETs identified in retrieved human thrombi (48, 49) provide biological context for PLR. Neither the ratio nor retrieved clot findings establish downstream microvascular thrombosis in an individual patient after MT.

Compared with NLR, evidence supporting PLR after MT remains limited (76–78). In Cao et al.’s cohort of 482 patients with successful angiographic reperfusion, day-1 PLR was independently associated with symptomatic intracranial hemorrhage (sICH), whereas its association with unfavorable 90-day functional outcome did not remain significant after Bonferroni correction (76). Broader MT cohorts (77, 78) should be distinguished from populations restricted to successful reperfusion. PLR is therefore best considered a candidate adjunctive marker rather than a standalone predictor; its incremental predictive value requires validation.

### 3.3 Composite inflammatory indices

Composite inflammatory indices attempt to capture the systemic inflammatory phenotype more broadly. The systemic immune-inflammation index (SII), calculated as platelet count × neutrophil count / lymphocyte count, combines platelet and neutrophil counts relative to lymphocyte count without directly measuring their activation. The systemic inflammation response index (SIRI), calculated as neutrophil count × monocyte count / lymphocyte count, incorporates monocyte count without directly measuring monocyte activation.

Direct MT evidence links SII measured at admission or early after MT to poor 90-day functional outcome (76, 77, 79). Chen et al. linked admission SII to the study-defined endpoint of “futile recanalization” (80). The association is more consistent for functional outcome than for symptomatic intracranial hemorrhage; notably, Han et al. found an association with poor functional outcome but no independent association with symptomatic intracranial hemorrhage (81).

Lattanzi et al. directly examined SIRI and futile recanalization in 184 patients with anterior-circulation AIS and successful EVT recanalization (TICI 2b or 3) (82). Higher admission SIRI was associated with 3-month mRS ≥ 3 after adjustment (OR 1.08, 95% CI 1.01–1.17, per unit increase). Discrimination was modest (area under the receiver operating characteristic curve [AUC] 0.644), and the retrospective, single-center findings require external validation. In a separate report of 885 successfully reperfused patients, Prandin et al. found associations with 24-h NLR and C-reactive protein (CRP); admission SIRI was associated with futile recanalization in the subgroup aged ≥80 years, but not in the overall cohort (83).

Findings for SIRI also vary by endpoint and population. In the successfully reperfused cohort reported by Cao et al., the association of day-1 SIRI with sICH remained significant after Bonferroni correction, whereas its association with 90-day functional outcome did not (76). Liu et al. evaluated admission SII and SIRI in 112 MT patients; neither was significant in the reported univariable logistic regression for 3-month outcome, despite between-group differences (78). That cohort was not restricted to successful reperfusion. Associations in other MT cohorts (79) and a broader AIS meta-analysis (84) should therefore be distinguished from direct evidence of futile reperfusion.

SII and SIRI combine several cell counts, but superiority over simpler indices has not been established across independently validated MT populations. A patient with neutrophilia, thrombocytosis, and lymphopenia may have a different risk profile from a patient with isolated neutrophilia. Composite indices could therefore be useful for phenotyping, but their formulas also amplify measurement variability and may be less intuitive for bedside decision-making. Other readily available measures, such as the C-reactive protein (CRP)-to-albumin ratio, have also been evaluated after MT but require further validation (85).

In another success-only EVT cohort of 101 patients, lower admission lymphocyte-to-monocyte ratio (LMR) was associated with 90-day mRS 3–6, whereas NLR and PLR were not significant in the multivariable model (86). This association also requires validation; the model included symptomatic intracerebral hemorrhage, a post-treatment complication.

### 3.4 Molecular biomarkers: IL-6, MMP-9, and endothelial injury markers

Blood-cell-derived ratios are attractive because they are inexpensive and available in routine care, but molecular biomarkers may offer information about selected pathways, although circulating concentrations are not direct measures of brain-specific activity. IL-6 is a central mediator of systemic inflammatory activation and has been associated with futile reperfusion after MT (69). A broader stroke meta-analysis also supports a prognostic association, but it is indirect for MT (87). In HIBISCUS-STROKE, 133 of 164 patients achieved successful reperfusion; 46 had a 3-month mRS score of 3–6. Higher IL-6 at 24 h from admission remained associated with this endpoint after multivariable adjustment (69). Lower admission IL-6 has also been linked to the first-pass effect, a procedural endpoint that should be distinguished from tissue perfusion and functional outcome (88).

MMP-9 is relevant to BBB injury, but its concentration alone does not establish barrier disruption or enzymatic activity. MT studies have associated peripheral MMP-9 with infarct growth and hemorrhagic transformation (66, 67). In a proof-of-concept study of 132 patients, Kollikowski et al. obtained paired blood samples from the collateral circulation distal to the occlusion before recanalization and from systemic arteries (68). Local MMP-9 was associated with major intracerebral hemorrhage and severe disability or death at discharge. These outcomes differ from 90-day futile reperfusion. Standardized assays, defined sampling sites and times, and validation in larger cohorts remain necessary.

Other molecular markers may eventually complement IL-6 and MMP-9, including markers of endothelial activation, glycocalyx shedding, complement activation, oxidative stress, and NET burden. The von Willebrand factor-cleaving protease ADAMTS13 (a disintegrin and metalloproteinase with a thrombospondin type 1 motif, member 13) has also been evaluated as a thrombo-endothelial marker of response to recanalization therapy (89). The main barrier is clinical implementation: many assays are not available rapidly enough to influence acute decisions, and cutoff values vary across platforms. The assays most likely to enter clinical practice are those that can be standardized and yield results within clinically meaningful time windows.

Recent MT-specific evidence broadens this molecular panel. In a prospective single-center cohort, ter Schiphorst et al. analyzed 139 patients with anterior-circulation LVO, successful reperfusion (mTICI at least 2b), and no reocclusion (90). Intracranial blood was sampled distal to the thrombus before recanalization; peripheral blood was collected immediately before and after treatment and at 24 h. Adjusted associations with 90-day mRS 3–6 were reported for vascular cell adhesion molecule-1 (VCAM-1) in intracranial and immediate postprocedural peripheral samples, interleukin-10 (IL-10) in preprocedural peripheral and intracranial samples, interleukin-15 (IL-15) before treatment, and serum amyloid A (SAA) immediately after treatment (90). These findings emphasize sampling source and timing, but do not establish causality or a validated clinical prediction tool.

Experimental studies offer possible explanations for these associations. VCAM-1 participates in leukocyte adhesion and was upregulated in ischemic cerebral vessels in rodents, but blocking it did not improve ischemic injury in that study (91). IL-10 regulates immune responses and can suppress inflammation, including detrimental interleukin-17A responses in experimental stroke (92). Higher IL-10 in patients with poor outcome could reflect a compensatory response, although this explanation remains a hypothesis. Astrocyte-derived IL-15 promoted cytotoxic lymphocyte responses and ischemic injury in experimental models (93). SAA, an acute-phase protein, activated microglial inflammasome signaling in cell and animal experiments (94). Circulating levels alone cannot identify the source tissue or show that these mechanisms occur in individual patients after MT.

Local vascular blood samples neither directly sample brain parenchyma nor measure capillary perfusion. Their interpretation depends on sampling location, collateral supply, timing, and procedural conditions. Invasive access also limits repeated sampling. Pairing local and peripheral samples with BBB or perfusion imaging may help assess biological specificity, but this approach needs prospective validation.

## 4 Inflammation as a potential contributor to futile reperfusion

Futile reperfusion describes poor functional outcome despite angiographic success. Angiographic reperfusion assesses restored flow in the downstream vascular territory, whereas clinical recovery requires preserved microvascular perfusion, viable penumbral tissue, an intact BBB, limited edema, and avoidance of hemorrhagic transformation. Inflammation may influence each of these downstream requirements.

Despite successful angiographic reperfusion after MT, downstream microvascular perfusion may remain impaired because of endothelial activation, leukocyte adhesion, platelet aggregation, capillary obstruction, and vasoconstrictive signaling. Experimental work supports neutrophil-mediated capillary obstruction (47), whereas NETs in MT-retrieved thrombi provide evidence from a different vascular compartment (48, 49). Complement activation and cytokine signaling can sustain endothelial dysfunction and impaired autoregulation (53, 95, 96).

We propose a conceptual, hypothesis-generating framework using lower- and higher-inflammation profiles. These are illustrative patterns along a heterogeneous and time-dependent spectrum, not clinically validated phenotypes. A lower-inflammation profile might coexist with preserved BBB integrity and adequate microvascular perfusion, whereas a higher-inflammation profile might accompany elevated systemic biomarkers, endothelial injury, barrier leakage, edema, or hemorrhagic transformation. These are possible relationships, not diagnostic criteria: systemic biomarker elevation does not establish local NET formation or no-reflow, and neither profile determines functional outcome. No validated classification thresholds or treatment-selection rules are established by this review.

Inflammation may be both a response to tissue injury and a contributor to its progression. Distinguishing these roles requires temporal and interventional evidence; an association after adjustment does not by itself show that inflammation determines tissue reperfusion.

The proposed framework is not intended to replace established predictors. Instead, we view it as a biological layer that may explain residual risk after accounting for age, NIHSS score, Alberta Stroke Program Early Computed Tomography Score (ASPECTS), collateral status, time, and final angiographic reperfusion grade. It may also explain why some patients with incomplete reperfusion recover better than expected, whereas others with near-complete reperfusion remain disabled.

The proposed profiles may be investigated for trial enrichment or added prognostic value when combined with clinical and imaging information. Such applications require prespecified definitions, reproducible assays and sampling times, external validation, and evaluation of clinical utility. Biomarker-guided care would additionally require evidence that acting on the classification improves outcomes. The framework currently provides no basis for changing monitoring intensity, blood pressure targets, antithrombotic treatment, or immunomodulatory treatment after MT.

## 5 BBB failure linking inflammation, futile reperfusion, and hemorrhagic transformation

Hemorrhagic transformation is one of the most clinically important complications after MT. Its risk reflects infarct volume, ischemic duration, reperfusion injury, blood pressure, thrombolytic exposure, procedural factors, and vascular integrity. Among these factors, BBB disruption provides a plausible mechanistic link between inflammatory activation and hemorrhagic complications (58, 60, 65).

The BBB is maintained by endothelial tight junctions, pericytes, astrocytic endfeet, extracellular matrix, basal lamina, and the endothelial glycocalyx. Ischemia–reperfusion damages this neurovascular unit through oxidative stress, cytokine signaling, neutrophil degranulation, NET formation, and matrix metalloproteinase-mediated proteolysis. Once barrier integrity is compromised, reperfusion exposes fragile microvessels to restored pressure and shear stress, increasing the probability of edema and bleeding.

BBB failure also contributes to futile reperfusion without overt hemorrhage. Increased permeability, endothelial swelling, microvascular compression, and loss of autoregulation can prevent effective oxygen delivery and accelerate secondary neuronal injury. Futile reperfusion and hemorrhagic transformation may share components of microvascular injury, but they remain distinct endpoints and neither establishes an inflammatory mechanism.

These outcomes can overlap without being interchangeable. Both may arise from the same injured neurovascular unit, with the clinical phenotype depending on the balance between microvascular obstruction, permeability, reperfusion pressure, infarct size, and repair capacity. A patient may therefore develop poor recovery through no-reflow without visible hemorrhage, through hemorrhage after reperfusion, or through both mechanisms simultaneously.

From a translational perspective, inflammatory biomarkers should be paired with imaging markers of BBB integrity. Dynamic contrast-enhanced magnetic resonance imaging (MRI), computed tomography (CT) permeability imaging, postprocedural perfusion imaging, and susceptibility markers of hemorrhage could help test whether biomarker changes are associated with barrier leakage or perfusion deficits after accounting for systemic inflammation. Their combined predictive and biological specificity require prospective validation.

## 6 Current challenges and future directions

### 6.1 Establishing causality beyond association

Most biomarker studies after MT are observational. Elevated inflammatory markers may contribute to reperfusion injury, but may also reflect larger infarcts, longer ischemic duration, systemic stress, infection, or comorbidity. Prospective studies need predefined sampling times, serial imaging, and careful handling of baseline severity, prestroke disability, reperfusion status, procedural factors, and treatment exposure. Residual confounding remains possible after statistical adjustment. The role of infarct growth or other post-treatment variables depends on timing and the question being studied; variables on a potential causal pathway should not automatically be treated as baseline confounders.

Studies differ in how they account for stroke etiology. Gao et al., for example, reported etiology, but their final model included age, hyperlipidemia, atrial fibrillation, baseline stroke and consciousness scores, and thrombectomy attempts rather than etiological classification (73). Adjusting for atrial fibrillation does not account for stroke etiology as a whole. Describing a cohort’s etiological distribution, including etiology in a model, and testing whether it modifies an association answer different questions. Reporting the distribution alone does not rule out etiological confounding.

Acute infection, particularly stroke-associated pneumonia, may also explain changes in CBC-derived indices over time. Studies should record when infection began relative to each blood sample and document relevant treatment exposure. Infection present before sampling may confound an association; infection developing later may be a mediator or an outcome, depending on the research question. Excluding infection at baseline or adjusting for in-hospital infection without recording its onset cannot fully resolve this ambiguity.

Causal inference will require more than larger cohorts. Relevant evidence includes temporal ordering, dose–response relationships, biological specificity, mediation through infarct growth or BBB disruption, and replication across independent datasets; observational patterns alone do not establish causality. Animal and human translational studies can also help identify whether modifying a pathway changes tissue reperfusion or merely changes a circulating marker.

### 6.2 Standardizing biomarker assessment and endpoints

Heterogeneity in sampling time, blood source, assay platform, cutoff values, patient selection, and outcome definitions limits translation. The illustrative sampling windows described in Section 3 require explicit timing relative to admission, stroke onset, or the procedure. Peripheral venous, systemic arterial, and intracranial samples should be distinguished. Futile reperfusion has also been defined using different angiographic thresholds, functional cutoffs such as mRS 3–6 or 4–6, and follow-up times. The operational definition used in this review does not replace those original definitions. Studies restricted to successful reperfusion must be distinguished from broader MT cohorts, and functional, hemorrhagic, imaging, and procedural endpoints should be reported separately. A shared minimum dataset should record these details, assay scales, effect estimates with uncertainty, and the covariates in the relevant model.

### 6.3 Integrating inflammation with multimodal prediction models

Inflammatory biomarkers alone are unlikely to replace established predictors such as age, baseline NIHSS, infarct core volume, collateral status, glucose, procedural time, and final angiographic reperfusion grade. Their best use may be as complementary biological variables in multimodal models that combine clinical features, angiographic parameters, perfusion imaging, BBB permeability imaging, and dynamic biomarker trajectories (70, 71, 76, 81). Model explainability does not establish that a predictor represents modifiable inflammatory injury; that interpretation requires separate biological and interventional evidence.

For prediction work, transparent reporting is essential. Candidate biomarkers should be prespecified, and missing data require explicit handling, including the loss of later samples through early death or clinical deterioration. Comparisons of serial models should use a defined prediction time and account for information already available at that time. Calibration must be reported, and external validation should include centers with different imaging protocols and treatment workflows. Without these steps, inflammatory prediction models may appear promising in derivation cohorts but fail at the bedside.

### 6.4 From biomarkers to targeted therapies

The ultimate goal is not only to predict futile reperfusion but also to prevent it. Potential therapeutic targets include neutrophil recruitment, NET formation, complement activation, IL-6 signaling, MMP-9 activity, platelet-immune interactions, endothelial glycocalyx preservation, and BBB stabilization. However, inflammation has time-dependent beneficial and detrimental roles: early excessive activation may amplify injury, whereas later immune responses may support debris clearance and repair (23, 29, 30, 97). From a therapeutic perspective, anti-inflammatory approaches will be most credible when treatment timing, patient selection, and safety monitoring for infection and hemorrhage are explicitly specified.

Evidence for candidate treatments comes from several settings. DNase I improved tPA-mediated lysis of retrieved human thrombi ex vivo (48, 49) by degrading extracellular deoxyribonucleic acid (DNA), rather than selectively preventing NET formation. In experimental cerebral ischemia, MBL inhibition with Polyman2 or anti-MBL antibodies reduced injury (53). Neither result establishes treatment efficacy after MT. An exploratory analysis of intravenous minocycline in AIS found lower circulating MMP-9 in patients treated with or without thrombolysis (98). Because minocycline has multiple actions, this finding alone demonstrates neither selective MMP-9 inhibition nor improved outcome after MT.

MIST-A directly examined adjunctive minocycline after successful MT (mTICI 2b/3) in anterior-circulation AIS (99). This multicenter, randomized, open-label, blinded-endpoint phase 2 trial randomized 189 patients, with 187 included in the modified intention-to-treat analysis. Oral treatment began within 1 h of recanalization. Minocycline did not significantly reduce the primary outcome of day-5 infarct growth ratio (adjusted geometric mean ratio 1.10, 95% CI 0.84–1.45). Ninety-day functional independence was also not improved (mRS 0–2: 68.1% versus 72.0%; adjusted risk ratio 1.00, 95% CI 0.70–1.43). Adverse-event rates did not differ significantly. These findings do not support routine adjunctive minocycline after successful MT, although the trial was powered for the imaging endpoint rather than definitive assessment of functional recovery.

EMPHASIS enrolled a broader AIS population: 1,724 patients were randomized within 72 h of onset to oral minocycline or placebo in a double-blind trial (100). The primary outcome, 90-day mRS 0–1, occurred in 52.6% versus 47.4% of participants with available outcome data (adjusted risk ratio 1.11, 95% CI 1.03–1.20). Serious adverse events did not differ significantly between groups. However, median baseline NIHSS was 5, and only 42 participants (2.4%) underwent endovascular treatment. The overall benefit therefore cannot be assumed to apply specifically after successful MT. Differences between the trials do not establish that reperfusion status modifies treatment efficacy, and neither trial validates a biomarker-based treatment-selection strategy (99, 100).

Clinical trial findings also depend on the endpoint assessed. In a randomized phase 2 trial of 108 patients undergoing endovascular treatment, tocilizumab, an IL-6 receptor inhibitor, reduced infarct growth from baseline to 72 h, the primary imaging endpoint (101). Definitive functional benefit has yet to be established. In a randomized trial of patients receiving thrombolysis with or without MT, the platelet glycoprotein VI inhibitor glenzocimab did not show an improvement in the primary endpoint of 90-day mRS 4–6 (102). Neither trial was restricted to patients with confirmed successful reperfusion or validated treatment selection based on the proposed inflammatory profiles. Targeting a platelet pathway alone does not show that treatment modifies platelet-immune interactions.

Future trials should distinguish prevention of peri-reperfusion injury from treatment after confirmed reperfusion, with explicit assessment of treatment timing, target engagement, functional outcomes, and infection or hemorrhage risk. Biomarker-guided therapies for futile reperfusion require prospective validation before clinical use (Figure 2).

**Figure 2:**
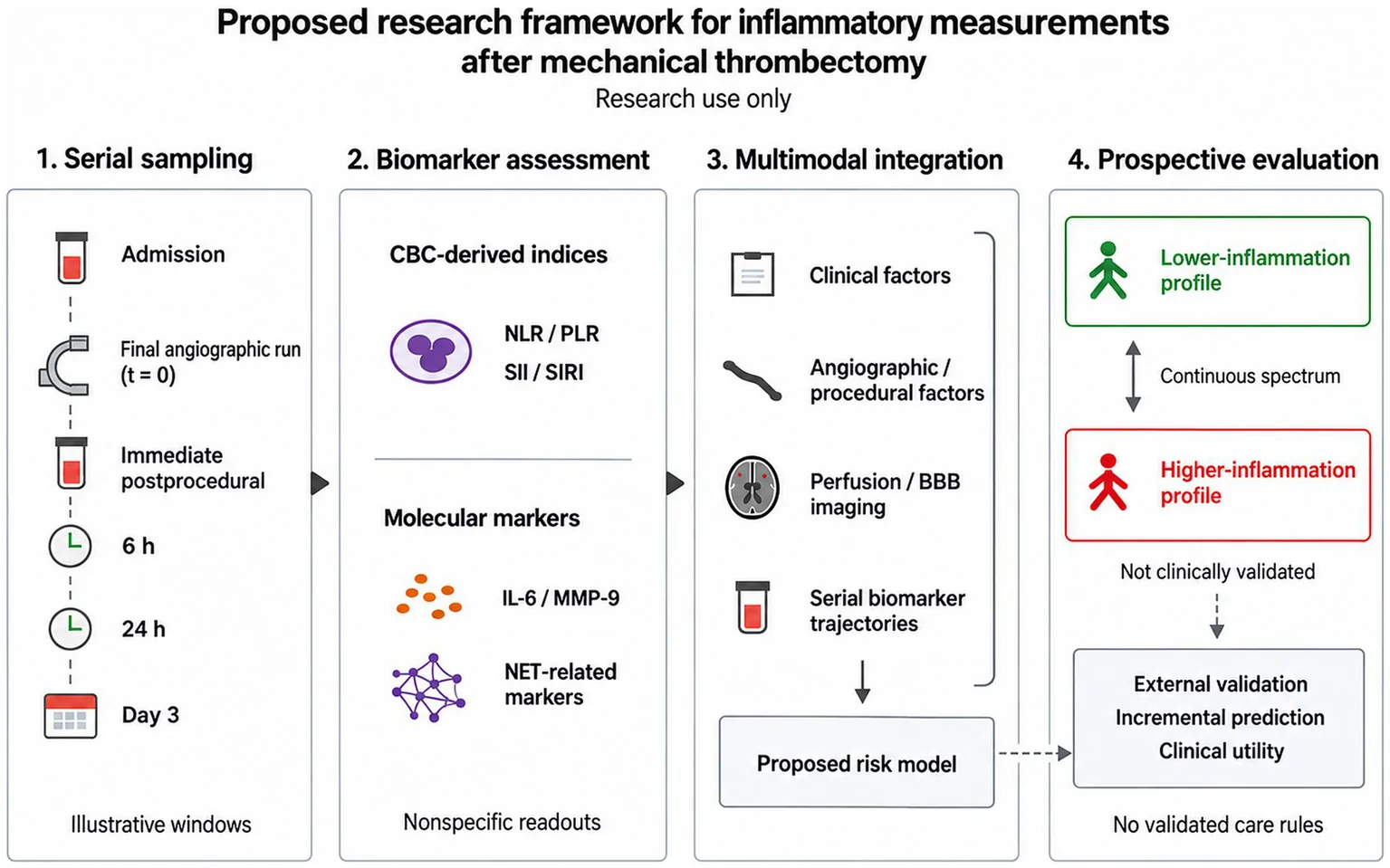
Proposed framework for studying inflammatory measurements after mechanical thrombectomy (MT). Admission, immediate postprocedural, 6-h, 24-h and day-3 samples illustrate a research schedule for which there is no clinical consensus. Postprocedural intervals start at the final angiographic run (t = 0); admission samples are not necessarily collected before reperfusion. These nonspecific measurements may reflect pre-existing inflammation, ongoing ischemia, reperfusion, evolving tissue injury, infection or other systemic complications. The model combines serial biomarkers with clinical, angiographic, procedural, perfusion and blood–brain barrier (BBB) information. Dashed arrows mark links that require external validation and prospective assessment of added predictive value and clinical utility. The lower- and higher-inflammation profiles lie on a continuous spectrum that changes over time. They have no validated thresholds and should not, on their own, determine monitoring intensity or treatment. NLR, neutrophil-to-lymphocyte ratio; PLR, platelet-to-lymphocyte ratio; SII, systemic immune-inflammation index; SIRI, systemic inflammation response index; IL-6, interleukin-6; MMP-9, matrix metalloproteinase-9; NET, neutrophil extracellular trap; CBC, complete blood count.

## 7 Conclusion

Mechanical thrombectomy has transformed treatment for acute ischemic stroke caused by large-vessel occlusion, but successful angiographic reperfusion does not invariably translate into neurological recovery. This persistent discrepancy argues against equating arterial reopening with functional recovery.

Collectively, current evidence indicates that inflammation may be a contributor to tissue injury after reperfusion. Ischemic inflammatory priming, reperfusion-triggered thrombo-inflammation, endothelial dysfunction, BBB disruption, and microvascular no-reflow may sustain tissue-level injury despite successful arterial reopening, and may contribute to poor functional outcome or hemorrhagic complications. The evidence includes model-specific mechanisms and clinical associations of differing causal strength.

Among currently available biomarkers, NLR has been repeatedly studied after thrombectomy, while SII, SIRI, PLR, IL-6, MMP-9, and other molecular markers remain promising but require further validation. We view inflammatory biomarkers not as isolated predictors but as nonspecific measurements that may complement clinical, imaging, and procedural assessments.

Future studies should test whether dynamic inflammatory profiles improve prediction beyond established variables and whether pathway-specific treatment can improve tissue perfusion and functional outcome without excess infection or hemorrhage.
